# Etiology and Pathology of Disease

**Published:** 1905-08

**Authors:** J. T. Roan

**Affiliations:** Quitman, Ga.


### ETIOLOGY AND PATHOLOGY OF DISEASE.

BY J. T. ROAN, M.D.,
QUITMAN, GA.

Since medical science can not be classed as an exact product of wisdom in perfection, we acknowledge to the world that medicine is not now, and probably never will be, an exact science. While it is true we may point with pride and say "See what wonderful strides it has made during the last quarter of a century," yet out in the unexplored fields of philosophy and etiological exactness we find many phenomena curious and puzzling. And we know other investigators, however painstaking they may have been, have only gone as far as it is possible for the sight of man to go with the use of powerful microscopes and other mechanical appliances. You will bear with me, inasmuch as pathology and etiology to most minds are tedious subjects, while I attempt to lay before your minds by the means of analogy and philosophy a clearer, broader and a surer foundation for the etiology and pathology of many of our common diseases, such as nearly all the fevers, wherein the living microbes have been discovered by the microscope, in those in which we know they do exist, the logical conclusions are that they are developed by the laws of life, each after its kind, that is, they are incubated and all of them alike come from an egg or ovote and are hatched by the same laws as other eggs by the days of the multiple of seven. Many of the smaller insect eggs are hatched into life in seven days, the smaller birds' eggs in fourteen days, the domestic fowls' eggs, such as the chicken, the guinea and many others, in twenty-one days; geese and peafowls in twenty-eight days. But taking note of this analogy, we readily comprehend that the smallest and the greatest of all eggs are hatched into life by this common law, upon the multiple of seven days. With this as my logical base I assert that all microbes and animolcules preying upon the system are developed from eggs. If we accept this to be the true hypothesis it will account for the stages of development in many of our diseases. It will also account for many of the so-called crisis days in many of our fevers. To illustrate, typhoid fever is marked, step by step, by these crisis days. Now the inquiry may come, if you accept this as the true etiology, how may it help us in healing such diseases and how help us to a more comprehensive practice? I will illustrate by inviting your attention to the authorities and practices that were in common use fifty and sixty years ago. The best authorities then approved of giving such treatment as sweating-teas, drastic purgatives, flebotany, and still further back in the history of medicine they approved the cruel regime of withholding liquids, especially water, from fever patients. The reasoning of the pathologists of that day and time was that such treatment reduced inflammation and congestion and drove out the poisonous substances from the system. In spite of such indefinite treatment many of their patients survived. Then came the more rational practice of elimination and stimulation; after all these came the hydropathist with water, hot and cold, with sheets, blankets and packs, proclaiming that water was the panacea for every ill. All the foregoing had some success, and it was in proportion as that their blind efforts eliminated the ovates before incubation. The reason why they gave such treatment was a commendable one; they said it was because it cured their patients. They reluctantly acknowledged that they did not know the etiology of such diseases. But I claim, according to my conception of the etiology of such diseases, that the reason is plain why they had any success at all, was that the eggs or ovates of such diseases were stimulated before they were incubated or hatched into living germs. By reviewing past uses in what was termed "Good Practice" even good was accomplished, though the practitioner did not know the true etiology of such diseases. In this I do not desire to place myself in the attitude of a critic of those faithful and able medical minds of the past. The marvel is how they accomplished their work as well as they did. We could not criticise the blind for not seeing the beauties of the rolling ocean, or observing the roughness of the highway. We now come to this good day in pathology and practice and find that the accepted authority is to destroy the microbe by aseptic medicines, such as the creosote derivatives, etc. This, I contend, is good practice as far as it goes, and is in harmony with my idea of etiology and pathology of these diseases, only I would add to this a perfect system of elimination. My object in this is to get rid of the ovates before they are hatched into living microbes. By the foregoing we see that it is very necessary that a physician should know and understand the etiology as nearly as possible of all diseases he is called upon to treat, and by having such knowledge his efforts will not be upon the ground of uncertainty, but upon the field of a more exact science. As to the ovates being the foundation of disease, per se, I will recapitulate by analogy and by illustration. We will suppose you have planned to make a journey of three or four thousand miles, accompanied by your entire household, say four or five.

We will suppose that they had been severally exposed to measles, mumps and whooping-cough during the last ten days and you do not want to be away from home till the danger of the developing stage of the disease had passed. You would likely consult your medical adviser about the matter, to know whether it would be safe to make the trip. Your doctor would look wise and knowingly and tell you mumps, measles and whooping-cough, as the case might be, developed upon the seventh, fourteenth and twenty-first days and would add that he had known some to develop even as late as the twenty-eighth or thirty-fifth day. He would be reciting some truisms that are accepted throughout the world. But probably he would have no conception of the true etiology of why these facts and truisms are so. And again you are called to treat a case of typhoid fever. It may be in a family where the mother and father are over-solicitous and must know a great many things from the attending physician, and they will ask when can we expect little Johnny or Willie to get worse or better, as the case may be. You will tell them about the fourteenth, twenty-first, twenty-eighth, or even as late as the the thirty-fifth or forty-second day. You will likely inform them that these particular days are the crises in this disease, in which they will get better or their fever will go higher. I will explain by my system of etiology why this is so, for if the patient should receive such treatment before these so-called crises days that will eliminate the eggs before the new crop or army has hatched into living microbes the fever will then go down. If not, the fever will go up or continue and the tendency will be worse and the chances of recovering less. By basing your treatment upon this idea of etiology of such diseases you will give aseptics to destroy the microbes that are already in the system and in full play, doing active mischief. You will also give such treatment as will promote elimination and support the system in order to get rid of the eggs before hatching and thus abort the disease at the so-called crises days.

We will now pass to a still more tedious part of our subject in etiology of such diseases as we have under consideration and investigate the laws of life that govern the habits of the living microbe and just how it is they produce the conditions we call disease. I know of no etiologist who has given a satisfactory solution to these puzzling phenomena. In this I ask your special indulgence, for I may be tedious and tiresome while I try to make something plain to your mind. Possibly you are familiar with the truism that nearly all fevers have what we know as periodicity, that is, they have their high and low periods once in twenty-four hours and fifty minutes (this is said to be the average). And this evidently is a truism and we naturally ask ourselves the question, why is this and what is the law that governs it? I have only to point you to some of the habits of these living microbes and you will apply or deduct the only logical answer.

The microbe is subject to the same common laws that govern the life and habits of other animals of the lower order of development. To illustrate, you inquire as I have done from men who follow fishing on the coast fisheries of the United States. They will tell you they have seen great multitudes and schools of fishes swarming in the ocean waters when the tide is flowing landwards, and at such times of the tide-flowing the fish will feed on anything, even one another, and at such times you can catch them as fast as you can bait your tackle and the bait need not be over-tempting. But as soon as the water or tides have started oceanwards the nature of the fish seemed to have changed. They will not feed upon anything, however tempting it may be, this phenomena always taking place upon the tides controlled by the moon, and this is the tide that comes once in twenty-four hours and fifty minutes (this is the average). This peculiarity of the fish is not a just happens so, but there is a law as I conceive and contend that governs their habits. This same law I contend is the cathode or anode electrical influence that governs the tides and is the same that has its direct influence over all lower animal life as well as over the tides. Then fish and microbes have a common time to feed and to sport in activity and a common time to be inactive. Therefore, I contend that this cathode or anode electrical force produces this law and all lower animal life, if not all life in common, is influenced and in a measure controlled by it.

I am well aware that three hypothesis has been entertained in etiology as to how the microbes accomplish injury to the system. It has been assumed by some able etiologist that it is a struggle for life for the mastery of the system, and those entertaining this view, contend that the microbes and the cells vie with each other over the alimentary materials carried by the blood and lymph as to which of the two could succeed in appropriating the most and reduce the other to starvation. Another authority, equally able, assumes a mechanical obstruction of the capillaries by microbe life, and still others of a more modern date, contend that it is an infectious process as an intoxication that the microbes contain and eliminate and thereby poison the system. All three of these etiological ideas have had their able advocates. All three, in some measure, are true. But let us make a broader and more searching survey for the truth, and we may find the foregoing conditions are only pathological conditions, following and pointing to the true etiology of such diseases as we have under consideration. And it is this that these millions and innumerable armies of microbes at or near the same time, feed upon the average, once in twenty-four hours and fifty minutes. And this feeding is done upon the periferal nerve endings. As you are aware, from your knowledge of physiology, that each flesh mesh or cell where the blood corpuscles pass from the end of an artery to the beginning of a vein. Just at this junction each one of these flesh cells are supplied with two very fine nerve (aferrent and efferent) endings, and it is upon these the microbes endeavor to satisfy their appetites. It is then the system feels the shock, and during and after the shock the brain has millions increased vibrations transmitted to it from the nerve periphery from all parts of the system, when microbes are feeding. It is a principle in philosophy, accepted and well founded, that energetic vibration is the sole cause of all heat. I do not affirm or assert this to be a fact, but to me it seems highly probable that this is the more reasonable cause of the increased heat or fever in the system. But I do contend that toxemia produces a morbid condition which follows the microbia shock or vibrations, and the toxemia is given off or excreted from the microbes. (In this I agree with other etiologists.) The increase heat or fever in the system consumes or burns out the tissues, while the microbes are lowering the nerve resistance. By taking this idea in etiology to be the true hypothesis, the plan of treatment naturally presents itself, and consists in supporting the nerve force and giving such other treatment as will eliminate microbes and eggs of the disease through the sweat glands of the skin, and having all other glands of the system judiciously at work carrying off the toxemias and unhatched eggs (this should be done without lowering the nerve force), at the same time, produce as near as possible aseptic and antiseptic conditions, both in the blood and tissues of the whole system. This will destroy the microbes, first by acting on them like an anesthetic, then complete torpor, after this the blood corpuscles tow them out like a tow boat, just as they do the dead tissues, and deposit them in the great scavenger gland (the liver) of the system, there to pass into the gall-bladder, thence through the ductus-communis choledochus into the alimentary canal and thence from the system.

While I do not assert it to be a fact, it is my opinion that the microbes are attached in the tuberiferis of the flesh cells during the time I specify as the feeding time, and at other times they are more likely to be found in other parts of the system—the spleen or bones, or in a torpid or dead state in the blood in circulation. And again it is asserted by others that they have been found in the gall bladder, and there become a nucleus for gall stone. Touching the subject of toxemia in the blood, my ideas of pathology and etiology agree with the able pathologists and etiologists who have done so much for the world of suffering humanity in the past. They, too, had to brook the criticisms of their associates and meet the scorn of those who adhered to the well-beaten paths in which many thousands of physicians had traveled and affirmed that their ideas of pathology and etiology were the last analysis of the long-hidden truth, and beyond it would be only folly and waste of time for others, however able, to consider. But as the years of the last century rolled by in single file, it was during this time that powerful microscopes were perfected and our thinking and reasoning etiologists were placed face to face with the hidden truth, and the critics and scoffers were made to see with mechanical eyes the truth for which they had so manfully contended by philosophy and analogy for over half a century. And it was then medicine and surgery received an impulse forward, and their progress has been marked by leaps, bounds and strides, until now no man or set of men would denominate them as cranks in medicine or the hoodoos in etiology, or demean them from that high standard which entitles them to the reverential respect and love of a grateful civilization. And were I asked to point to the field in which the greatest achievements for human good would be made during this century, with selfish pride I would give it as my opinion and point you to etiologists, the continued thinkers and undervalued workers of our profession. And were I asked what should be the most important subject that should engage the thoughtful medical minds of this country, I would say teach the young medical men if they would be useful and great, to harness their faculties of mind, heart and soul to plow well and deep the fields of etiology and pathology, and know well what other men on each and every subject think and teach. But be sure to analyze the subjects well within your own mind and have your mental approval, for this is the great highway to true usefulness and greatness of intellect; for he that prolongs the life of his fellows or assuages the pain and suffering of humanity is great indeed, and should be more esteemed than Napoleon, Alexander or Caesar, who each in his time filled Europe and the world with carnage, blood, sorrow and tears, and left the world in sackcloth and ashes.

Not because of their leaving did the world moan, but because they had bequeathed to it mountains of suffering and pain and rivers of blood and tears, and upon these documents of bequeathment were written all over them with their ingratitude to God and man and the memory of man's inhumanity to man. I hope to be pardoned for this slight digression from my subject, as it is not my intention to enter into an eulogistic discourse, but I do commend to your minds the ideas that may be new to you, and possibly not in accord with the so-called and accepted authorities on etiology and pathology.

And should it be necessary to offer an apology for what I have endeavored to lay before you for your philosophical and painstaking researches, I would point you to the words of truth enunciated by the great Burke, "A man who works beyond the surface of things, though he may be wrong himself, yet he clears the way for others, and may chance to make even his errors subservient to the cause of truth."

May 26, 1905.

---

It is time the physicians of this country called a halt. Month after month we are literally deluged with a multitudinous number of new and needless remedies—remedies that only find their need in the financial profit of the manufacturer. Were it not for the help of thoughtless physicians this would have long ago been brought to a stop. Not that we decry the work of the scientific chemist in his search for new methods and new drugs, for we all feel his need and acknowledge our depth of gratitude to his labors in the past, but this exploitation of various combinations of well-known drugs, and of those which have not received the seal of scientific approval ought to stop. It is only the ready sale procured that encourages this industry. If physicians would study carefully the National Formulary there would be less need of combinations of medicines put up by "manufacturing" chemists. Any good druggist can put up a National Formulary prescription as well as any one else. Show us any preparation that has a large sale on account of its supposed good properties and we will show you its analogue in the National Formulary, which will have the identical effect.—*Medical Recorder.*

